# Health-Related Quality of Life and Unmet Healthcare Needs in the First Year Following Moderate-to-Severe Traumatic Injuries—An Observational Study

**DOI:** 10.3390/jcm14124213

**Published:** 2025-06-13

**Authors:** Joanna N. Selj, Paul B. Perrin, Solveig L. Hauger, Cecilie Røe, Håkon Ø. Moksnes, Christoph Schäfer, Vilde M. Danielsen, Torgeir Hellstrøm, Nada Andelic, Mari S. Rasmussen

**Affiliations:** 1 Department of Physical Medicine and Rehabilitation, Oslo University Hospital, N-0424 Oslo, Norway; joselj@ous-hf.no (J.N.S.); cecilie.roe@medisin.uio.no (C.R.); hakmok@ous-hf.no (H.Ø.M.); christoph.schafer@unn.no (C.S.); vidani@ous-hf.no (V.M.D.); uxhetz@ous-hf.no (T.H.); nadand@ous-hf.no (N.A.); 2 Institute of Clinical Medicine, Faculty of Medicine, University of Oslo, N-0318 Oslo, Norway; 3 School of Data Science and Department of Psychology, University of Virginia, Charlottesville, VA 22904-4101, USA; pperrin@vcu.edu; 4 Department of Research and Innovation, Sunnaas Rehabilitation Hospital, N-1453 Nesoddtangen, Norway; solveig.hauger@gmail.com; 5 Department of Psychology, Faculty of Social Sciences, University of Oslo, N-0373 Oslo, Norway; 6 Department of Rehabilitation, University Hospital of North Norway, N-9038 Tromsø, Norway; 7 Department of Clinical Medicine, Faculty of Health Sciences, UiT The Arctic University of Norway, N-9037 Tromsø, Norway; 8 Research Centre for Habilitation and Rehabilitation Models & Services (CHARM), Faculty of Medicine, Institute of Health and Society, University of Oslo, N-0374 Oslo, Norway; 9 Faculty of Health Sciences, Oslo Metropolitan University, N-0166 Oslo, Norway

**Keywords:** health-related quality of life, trauma rehabilitation, unmet needs

## Abstract

**Background:** Traumatic injuries often lead to long-lasting impairments and complex rehabilitation needs. Trauma patients report lower health-related quality of life (HRQoL) and increased needs for healthcare and social support. This study aimed to describe HRQoL trajectories in a Norwegian cohort with moderate-to-severe trauma over 12 months and assess the relationship between unmet needs and HRQoL. **Methods:** A multicenter prospective cohort study with follow-up at six and twelve months post-injury included adults aged 18+ with a New Injury Severity Score (NISS) > 9 and a minimum hospital stay of two days. HRQoL was assessed using the EQ-5D-5L. Needs and unmet needs were evaluated using the Needs and Provision Complexity Scale. Hierarchical linear modeling (HLM) examined predictors of HRQoL trajectories. **Results:** Of 538 participants, 83% were followed up at six and/or twelve months. Mean age was 52 years, falls were the most common cause of injury (44%), and 76% had a severe injury (NISS > 15). HRQoL remained stable, except for improvements in usual activities and anxiety-depression domains. HLM showed that older age (*b* = −2.698), longer hospital stay (*b* = −4.108), and unmet healthcare (*b* = −1.094) and social support needs (*b* = −3.132) were associated with worse HRQoL over time. Unmet personal care needs were linked to improved HRQoL (*b* = 2.654). The only significant predictor*time interaction was between unmet healthcare needs and overall HRQoL. **Conclusions:** HRQoL largely remained stable, with improvements in some domains. Unmet healthcare needs predicted a decline in HRQoL, highlighting the importance of timely support and targeted interventions from health professionals.

## 1. Introduction

Traumatic injuries, defined as physical injuries with sudden onset and severity that require immediate medical attention, constitute a significant public health concern globally [1,2]. Such injuries can result in long-lasting physical, cognitive, and mental impairments that disrupt daily activities, occupational performance, family functioning, and social participation, ultimately reducing health-related quality of life (HRQoL) [3,4,5,6]. Improved emergency management has increased survival rates following severe traumatic accidents [7], underscoring the need to evaluate outcomes and the impact of trauma on quality of life.

The extent of injury-related difficulties depends on several factors, including injury severity, pre-injury sociodemographic characteristics, comorbidities, and post-injury factors, such as the availability of healthcare services [8]. Severely injured patients with multiple traumas report lower HRQoL compared to population reference groups [6]. However, studies show variability in the relationship between injury type, severity, and HRQoL. For instance, spinal cord injuries, hip fractures, and lower extremity injuries are associated with diminished long-term functioning—an increasingly acknowledged component of population health [5]. Notably, while HRQoL is typically reduced in severely injured patients with multiple traumas, those without intracranial injuries often exhibit improved long-term HRQoL [9,10].

Time since injury is an important factor influencing HRQoL after trauma. A systematic review indicated that HRQoL improves during the first year post-injury [11]. Several studies suggest that both physical and mental health improve over time following a traumatic accident [12,13,14]. The SF-36, EQ-5D-5L, and WHOQOL, are among the most used HRQol measures and emphasize physical function and mental health. In this study, the EuroQol-5 Dimensions-5 Levels (EQ-5D-5L) was used to assess mobility, self-care, usual activities, pain, and anxiety and depression, covering important aspects of both physical and emotional well-being. Studies using the EQ-5D demonstrated improvements in usual activities but also highlighted persistent pain and mental health concerns in multi-trauma patients [11]. In addition, optimism and lower baseline levels of depression and posttraumatic stress are key predictors of better HRQoL at one year, highlighting the importance of psychological factors in recovery [15]. Nevertheless, physical function and pain-related HRQoL remain significantly impaired in the trauma population compared to the general population [15]. These findings underscore the need for enhanced healthcare and rehabilitation services to support recovery and improve HRQoL outcomes for trauma survivors.

Despite the understanding that patients with physical trauma require multidisciplinary healthcare and rehabilitation to improve functioning and thereby HRQoL, there is a paucity of studies investigating the relationship among healthcare provision, rehabilitation service delivery, and HRQoL. By using the Needs and Provision Complexity Scale (NPCS) [16], a previous prospective multicenter cohort study found that patients with moderate-to-severe trauma treated in a trauma center experienced unmet needs for community-based healthcare and social support in the first year post-injury, indicating that many patients do not receive necessary care [17,18]. Predictors of unmet community-based service needs included age, pre-injury comorbidity, geographic living area, profound injury severity, severe head injury, and higher disability levels [17,18]. To the authors’ knowledge, no study has specifically investigated the relationship between unmet healthcare and social support needs as assessed by NPCS and HRQoL in the trauma population. Thus, our study adds value by incorporating the concept of unmet needs and illustrating how these needs influence HRQoL trajectories. Such knowledge can inform healthcare service planning and guide service improvements in well-being and HRQoL, which are key objectives of health interventions and rehabilitation efforts.

The purposes of this study were as follows:

1. To describe the trajectory of HRQoL in a Norwegian cohort of patients with moderate-to-severe trauma during the first 12 months post-injury.
2. To assess the relationship between unmet needs for healthcare and social services and HRQoL at six and twelve months post-injury, controlling for sociodemographic and injury-related variables.

## 2. Methods

### 2.1. Setting and Participants

This multicenter, prospective cohort study included follow-ups at six and twelve months post-injury and identified unmet rehabilitation needs in the first year following trauma [17,18]. In this study, we enrolled adult patients with moderate-to-severe traumatic injuries admitted to the regional trauma centers at Oslo University Hospital (OUH) in southeastern Norway and the University Hospital of North Norway (UNN) in the north. Recruitment periods were from January 2020 to December 2020 (OUH) and from February 2020 to January 2021 (UNN). Eligible patients were enrolled during their hospital stay or immediately after discharge from the trauma care unit. A total of 538 adult patients consented to participate. We analyze data gathered at baseline, as well as at the six- and twelve-month follow-up points.

### 2.2. Inclusion and Exclusion Criteria

Adults aged 18 years or older admitted directly or within 72 h to one of the two regional trauma centers with a moderate-to-severe traumatic injury, indicated by a New Injury Severity Score (NISS) greater than 9 and a minimum hospital stay of two days were evaluated for eligibility. The study excluded non-Norwegian residents and patients with limited proficiency in Norwegian or English. The NISS was utilized because it provides a more accurate assessment of injury severity and better identifies major trauma compared to the Injury Severity Score (ISS) [19]. The National Institute for Health and Care Excellence recommends that patients in a trauma center with an ISS above nine undergo evaluation for rehabilitation needs [20].

### 2.3. Procedures

Patients were identified through daily trauma report meetings, supplemented by lists of new trauma hospitalizations, and searches in the hospital’s medical record system for trauma-related admissions. Eligible patients and/or their caregivers received oral and written study information in person during admission or via telephone shortly after discharge. Informed consent was obtained from all participants. Written consent was obtained either during the initial meeting or sent by post to those contacted after discharge. Outcome data were obtained from medical records, hospital-based trauma registries, and self-reports from patients or caregivers using questionnaires and interviews [21].

### 2.4. Main Outcomes and Definitions

The main outcome was health-related quality of life (HRQoL), as assessed by the EuroQol 5-dimension 5-level questionnaire (EQ-5D-5L) at six and twelve months post-injury [22,23]. The EQ-5D is a standardized, generic self-report tool designed to assess health outcomes, consisting of two parts. The first comprises five dimensions of health: mobility, self-care, usual activities, pain/discomfort, and anxiety/depression. Each dimension is rated on a five-point severity scale: no problems (1), slight problems (2), moderate problems (3), severe problems (4), or unable/extreme problems (5) [23]. The scores for these five dimensions can be presented in health profiles or combined into a global health index reflecting overall HRQoL, where higher values indicate poorer outcomes. Part two is a Visual Analogue Scale (EQ VAS) ranging from 0 (worst health state) to 100 (best health state), often used as a general measure for HRQoL.

### 2.5. Data Collection and Variables/Independent Variables

Sociodemographic and injury-related variables were collected from baseline data, including medical records, hospital trauma registries, and information provided by the patient or caregiver. Sociodemographic data included age, sex, living status, education, geographical centrality of living, and pre-injury comorbidities. Living status was dichotomized into “partnered” and “single/lives alone”. Education was classified as elementary school, high school, or university. Geographical centrality of the patient’s municipality of residence was categorized using the Norwegian Centrality Index (NCI), indicating its level of centrality, with values 1–2 classified as central and 3–6 as less central areas. Pre-injury comorbidity was assessed using the American Society of Anesthesiologists Physical Status (ASA-PS) classification [24] and medical records of pre-injury diagnoses, including mental health and substance abuse. The ASA PS scores were dichotomized into healthy (ASA-PS score 1) and systemic disease (ASA-PS scores 2–4, with no scores exceeding 4).

Injury-related variables encompassed injury severity and the number of injuries, extracted from the trauma registries (OUH, UNN) using Abbreviated Injury Score (AIS), NISS and ISS. The AIS is an anatomical scoring system that classifies injury severity by body region [25]. The ISS and NISS are based on the AIS scoring and provide scores of overall injury severity. For this study, NISS of 10–15 were defined as moderate, and scores of 16–75 as severe. Length of stay (LOS) was measured by the number of days in the acute care departments of the trauma centers. Discharge destinations were categorized into four groups: home, local hospitals, nursing homes/others, or specialized rehabilitation in a hospital or institution within the specialist healthcare system.

Unmet rehabilitation needs were assessed by comparing the estimated needs for healthcare services, rehabilitation, and social support at the time of discharge from the trauma department and at the six-month follow-up with the actual services provided during the first six months (0–6 months) and the subsequent six months (7–12 months) post-injury, as reported by patients and/or caregivers. To assess the extent of unmet needs, we used the Needs and Provision Complexity Scale (NPCS) [16]. The NPCS is a 15-item tool assessing met and unmet needs in community-based healthcare, social care, and rehabilitation (range 0–50). The clinician version evaluates an individual’s needs for rehabilitation and support (NPCS-Needs), while the patient version captures the services received (NPCS-Gets). Its two domains are Health and Personal Care Needs (subscales: Healthcare [0–6], Personal Care [0–10], Rehabilitation [0–9]) and Social Care and Support Needs (subscales: Social and Family Support [0–13] and Environment [0–12]). The NPCS has been validated in a Norwegian population comprising individuals with TBI and aneurysmal subarachnoid hemorrhage (aSAH) [26]. The clinician-rated NPCS demonstrated good to excellent inter-rater reliability, while the patient-reported version showed good to excellent test–retest reliability. Absolute agreement ranged from moderate to excellent across all clinician- and patient-rated items. A discrepancy score between NPCS “Needs” and “Gets” indicates unmet or exceeded needs, where a positive score indicates unmet needs. Rehabilitation specialists involved in the study were not part of the clinical teams providing care to the participants. They estimated the NPCS-Needs based on review of medical records, supplemented by their professional judgment and clinical expertise. The evaluation considered several factors, including the type and severity of injury, documented impairments, functional assessments conducted during the acute phase, as well as the patient’s age, premorbid functional status, and living arrangements. At six months, patients reported their symptoms and functioning, as well as their assessment of the level of services received during the first six months post-injury. The patient NPCS-Gets was collected via telephone interviews conducted at six- and twelve-month follow-ups [18].

### 2.6. Statistical Methods

All statistical analyses were conducted using SPSS software, version 30 (IBM SPSS, Chicago, IL, USA). Descriptive statistics were calculated to describe the study sample and outcomes. For normally distributed data, means and standard deviations (SDs) were reported, while medians and interquartile ranges (IQRs) were reported for non-normally distributed data. Categorical variables were presented as frequencies and percentages. Paired-samples *t*-tests and chi-squares were used to examine changes in HRQoL and unmet needs over time.

Hierarchical linear modeling (HLM) was used to examine baseline predictors of HRQoL trajectories across six and twelve months after injury. Six separate main effects HLMs were conducted, with each examining the trajectory of a specific HRQoL domain: overall HRQoL, mobility problems, self-care problems, usual activities problems, pain-discomfort problems, and anxiety-depression problems. In all six models, the outcome variable was the respective HRQoL domain. Predictors were entered simultaneously as fixed effects, with continuous variables centered and categorical variables assigned a reference value of 0, along with time. All predictors were z-transformed (standardized) to allow for direct comparison of each parameter estimate.

For each HRQoL domain trajectory, the HLM assessed whether the overall height of the linear trajectory across the two time points could be predicted by demographic and injury-related factors. These factors included time (0 = 6 months, 1 = 12 months), sex (1 = man, 0 = woman), living status (1 = partnered, 0 = single), geographical centrality (1 = less central, 0 = central), age, injury severity, number of injuries, length of stay (in days), and unmet needs at the six-month follow-up (1 = need unmet, 0 = need met) in healthcare, personal care, rehabilitation, social-family, and environmental needs. For each predictor, an α of 0.05 was used to reflect statistical significance. To evaluate whether changes in HRQoL over time (i.e., differences in slope) varied based on the predictors, a second set of six HLMs was conducted—one for each HRQoL domain. These models included the same predictors as the main effects models, as well as time and interaction terms between time and the predictors. Given the lack of directional hypotheses regarding interaction effects, a Holm–Bonferroni correction was applied to the interaction effects across all models (e.g., the smallest *p*-value was evaluated at α = 0.050, the second smallest at α = 0.025, the third smallest at α = 0.017, the fourth smallest at α = 0.013, etc.).

## 3. Results

A total of 538 adult patients were included in this study, of whom 446 (83%) were followed up at least once, either at six and/or twelve months post-injury. At the six-month follow-up, the dropout rate was low (7.4%), whereas it was just above 20% at the 12-month follow-up. Dropout analyses showed that non-responders at the six- and twelve-month follow-ups were slightly younger than responders (43 years for non-responders vs. 48 years for responders, *p* = 0.05). No statistically significant difference was found in gender distribution between responders and non-responders.

**Figure 1:**
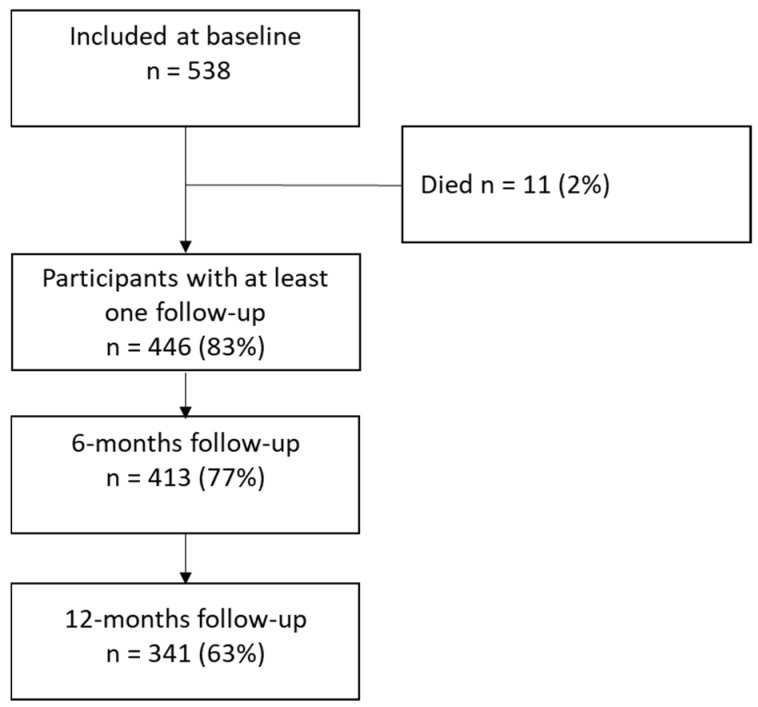
Flow chart.

The mean (SD) age of the 446 patients who were followed up was 52 (17.8) years, see Figure 1. Socio-demographics, injury, and clinical characteristics at baseline are presented in Table 1. Most patients (64%) lived with others (partners) and 58% resided in central areas. Approximately half of the patients were classified as healthy according to their ASA-PS score at the time of injury.

The majority of patients had sustained injuries from falls (44%) and transportation accidents (39%). The mean NISS was 24.2 (SD 12.0) indicating profound injury severity. Most patients had experienced multiple traumas, with a mean of six injuries (SD = 3.7). One-third of the patients had been discharged home following the acute hospital-stay, while the remaining patients had been discharged to other hospitals (43%) or specialized rehabilitation (20%). Only 2% had been discharged to nursing homes.

The mean NPCS-Needs total score estimated at baseline was 9.5, ranging from 0 to 34, see Table 1. The highest mean values were found in the healthcare and rehabilitation subscales.

### 3.1. Overall HRQOL and the Level Scores for Each of the Five EQ-5D Dimensions at Six and Twelve Months

Overall self-reported HRQoL measured by EQ-5D VAS showed no significant difference between six and twelve months (69.57 vs. 70.31, respectively). The domains of usual activities and anxiety and depression improved significantly in the same period, while the other domains remained unchanged (see Table 2).

### 3.2. Unmet Needs for Healthcare and Social Support Services at Six and Twelve Months

At six months post-injury, 54% of the patients had unmet needs, assessed by the NPCS total score, which decreased to 35% at the 12-month follow-up. The proportion of unmet healthcare needs increased slightly from 21% at six months to 27% at 12 months post-injury. Unmet needs significantly decreased from six to twelve months in the subscales of rehabilitation and social support. (see Table 3).

The internal consistency and reliability of the EQ-5D were assessed using Cronbach’s alpha at each follow-up time point. The Cronbach’s alpha coefficients for the EQ-5D across dimensions were 0.73 and 0.78, respectively, indicating satisfactory internal consistency. Similarly, the Cronbach’s alpha for the NPCS baseline estimation of need was 0.82, also reflecting a satisfactory level of reliability.

### 3.3. The Relationship Between Unmet Needs and HRQoL

#### 3.3.1. Main Effects Models

All statistically significant and non-significant fixed effects with standardized parameter estimates from the six main-effects HLMs and their *p*-values are presented in Table 4. For reference, the 95% confidence intervals of these parameter estimates are shown in Supplemental Table S1 in Supplementary Materials.

Factors associated with higher EQ-5D pain trajectories included being partnered, lower injury severity (as measured with NISS), a higher number of injuries, and living in less central regions. A greater number of injuries also predicted higher anxiety–depression trajectories. Older patients experienced lower overall HRQoL and higher mobility and self-care problem trajectories. Longer hospital stays predicted lower overall HRQoL and higher problem trajectories in all EQ-5D dimensions.

Patients with unmet healthcare needs had lower overall HRQoL trajectories and higher trajectories of EQ-5D anxiety–depression. In contrast, patients with unmet social-family needs had lower overall HRQoL trajectories and higher trajectories of problems related to usual activities. Conversely, unmet personal care needs correlated with higher overall HRQoL and fewer problems in the usual activities domain, while unmet environmental needs increased the trajectories of pain-discomfort problems.

#### 3.3.2. Interaction Effects Models

**Figure 2:**
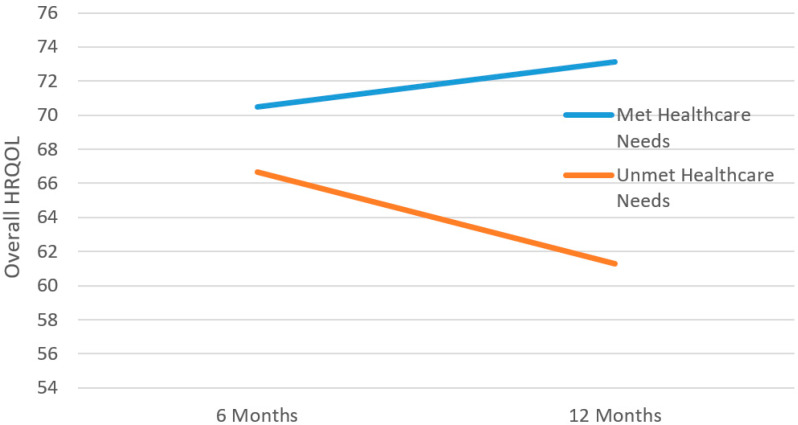
Main effect and time interaction of baseline unmet healthcare needs on overall HRQOL trajectories.

All statistically significant and non-significant interaction effects with standardized parameter estimates from the six predictor*time interaction effects HLMs and their *p*-values appear in Table 5. After applying a Holm–Bonferroni correction, the only significant predictor*time interaction effect identified was between unmet healthcare needs and overall HRQoL. These findings indicate that patients with unmet healthcare needs at six months experienced a decrease in overall HRQoL across time, while those with met healthcare needs appeared with an increase (Figure 2).

## 4. Discussion

This study describes the trajectories of HRQoL, as assessed by the EQ-5D in the first year after moderate-to-severe traumatic injury and their relationship with unmet needs for health, rehabilitation, and social support services.

In this study, the self-reported Visual Analogue Scale (VAS) score represents overall HRQoL. A decline in overall HRQoL, as indicated by a negative coefficient for the VAS score, reflects poorer perceived health. In contrast, higher scores (positive coefficients) in the individual EQ-5D domains reflect greater difficulties or impairments in those specific areas, and therefore worse HRQoL. It is also important to recognize that participants may perceive their overall health more positively than what is captured in their ratings across individual domains. HRQoL and its domains remained stable for the overall study sample from six- to twelve-month follow-ups, except for the usual activities and anxiety–depression domains, where patients reported significantly fewer problems one-year post-injury. This aligns with the findings of the systematic review by Silverstein, Higgins [11], which noted improvements in usual activities during the first year after trauma. A population-based prospective cohort study on more than 2000 trauma survivors in Australia found that the usual activity items of the EQ-5D were the only domain that continued to improve during the first three years after injury [27]. Additionally, a stable and resilient recovery pattern has been identified as the most common trajectory after physical traumatic injuries [28,29]. Resilience and ability to adapt may help to explain the reduction in emotional distress, such as symptoms of anxiety and depression as measured by the EQ-5D, during the first-year post-injury.

Of the sociodemographic factors, higher age predicted lower HRQoL trajectories and more problems in mobility and self-care. This has also been demonstrated in other studies [27,30]. However, Gross, Morell [30] noted that the age effect on HRQoL was mainly evident in those above 80 years. In our study, ages ranged from 18 to 93 years. A possible explanation for our findings is that older adults more frequently had comorbid conditions compared to younger individuals, leading to prolonged recovery times. This could have affected overall HRQoL, especially in the mobility and self-care domains. Previous research has shown that morbidity and reduced mobility negatively influence HRQoL in persons above 55 years [31,32].

While having a more severe injury (classified by a NISS > 15) predicted lower EQ-5D pain trajectories, having a higher number of injuries predicted higher EQ-5D pain trajectories. Pain is a common consequence of traumatic injuries, and a previous systematic review supports our findings of less severe injuries and multiple traumas are predictors of higher levels of pain [33]. Furthermore, studies have demonstrated that having multiple and orthopedic injuries negatively affects HRQoL, particularly in the areas of pain and discomfort [11]. This was evident in our results, where a higher number of injuries predicted higher pain and anxiety-depression trajectories. Contrary to our results, Angerpointner, Ernstberger [34] found that patients who sustained a severe injury (ISS > 16) had more impaired HRQoL compared to those with less severe injuries. We also found that length of stay, reflecting a more severe injury or other injury-related complication and/or comorbidities, was associated with reduced overall HRQoL and more problems in all EQ-5D domains. Pain and length of stay have been identified as important predictors of loss of independence [35]. It is important to identify these injury-related factors early and provide necessary services, as this can possibly reduce long-term pain and emotional distress. Additionally, having unmet environmental needs was associated with higher pain trajectories. Although environmental needs were among the least needed services in this study, implementing adaptations for patients with functional limitations affecting activity and participation is essential.

A significant proportion of trauma survivors experience persistent physical, psychological, and emotional consequences that affect daily life activities, work/study, and family responsibilities [4,11]. Many require acute, post-acute and long-term complex rehabilitation services, as recommended in the Norwegian National Trauma Plan [36] and a recent systematic review on multi-trauma rehabilitation [37]. Despite this, we have previously demonstrated that many individuals did not receive the services estimated to be necessary during the first six months post-injury [18]. This was particularly true for rehabilitation services (52% having unmet needs) and for social and family support services (50% having unmet needs).

While other studies have identified factors influencing HRQoL after trauma, no previous study has explored the influence of unmet healthcare needs on HRQoL trajectories. Our results demonstrated that unmet healthcare needs predicted lower HRQoL trajectories in the first-year post-injury. Furthermore, patients with unmet healthcare needs at six months showed a decrease in overall HRQoL. The Healthcare subscale of NPCS includes medical care and skilled nursing. During the first year after trauma, patients have several concerns, including physical health and medical issues [38]. Additionally, we found that having unmet healthcare needs was associated with higher trajectories of depression and anxiety. Research has shown that psychological consequences, such as symptoms of depression and anxiety, are common following traumatic injury. An Australian study on 201 trauma patients found that over half reported above normal levels of anxiety and/or depression during the first six months post injury [39]. After the acute hospital stay, most patients are discharged to their homes, sometimes without further scheduled follow-up, except for potential check-ups at the hospital. Having unaddressed questions about physical health or medical concerns might negatively impact HRQoL. The categories within healthcare needs also merit further discussion, as they provide a detailed framework for understanding specific areas where patients may require more focused interventions. These findings underscore the complexity of post-trauma rehabilitation and the necessity for tailored approaches that address both immediate and long-term patient needs.

Unmet personal care needs predicted higher overall HRQoL trajectories and lower usual activities problems. Personal care needs include care in and around the home, and requiring a personal assistant [16]. This counterintuitive result warrants further investigation to understand the underlying factors contributing to this phenomenon. We have previously demonstrated that at a six-month follow-up, the lowest proportion of unmet needs were within personal care and environmental needs [18]. This trend was also observed at the one-year follow-up [17]. Thus, many patients had their needs for personal care met within the first year after the injury. One possible explanation could be that people that did not receive personal care, especially formal care, perceived themselves as more independent.

The only significant predictor by time interaction effect was with unmet healthcare needs on HRQoL. Persons with unmet healthcare needs experienced a decline in HRQoL over time, while the opposite was evident for those having their healthcare needs met. Reasons for unmet needs may include personal reasons and access or availability to the needed services. Availability and quality of care are important predictors of HRQoL, and studies have demonstrated that people who are satisfied with the care they receive are more likely to adhere to treatment and show positive health behavior [40]. Thus, it is important to bridge the gap between the perceived needs and the services available. We have previously demonstrated in nearly the same study sample that half of the patients had persistent disability at 12 months after injury [8]. Considering this, many of these patients will likely need coordinated multidisciplinary services during the first year after injury [37].

In this study we investigated the relationship between healthcare service needs and HRQoL. However, it should be acknowledged that many factors not examined in this study may influence HRQoL trajectories. For example, Tøien, Bredal [15] found that optimism was an independent predictor of good HRQoL one year after injury. Levels of resilience and patients’ coping strategies could influence HRQoL and potentially also to what degree they seek formal healthcare services. Nevertheless, the results of this study highlight some of the needs of this patient group in the first year after injury and provide insight into the consequences of inadequate provision of formal services.

### Strengths and Limitations

The healthcare needs assessment in this study provides crucial insights into patient outcomes following trauma. Despite some attrition, the dropout rate in this study was low compared to other longitudinal cohort studies [41], enhancing the reliability of the findings. However, the methods used for the estimation of rehabilitation needs have not been extensively validated in a trauma population. This study relied solely on the estimations of rehabilitation specialists regarding the NPCS at the time of discharge from trauma centers, along with assumptions that appropriate ongoing services would be provided, and patient-reported telephone interviews at six and twelve months to assess received services. This approach presents challenges in accurately assessing rehabilitation needs, particularly given the dynamic nature of patient recovery over time. The NPCS completion by clinicians may have introduced subjectivity or variability depending on individual judgment. Moreover, the accuracy of the NPCS ratings was dependent on the availability and quality of information within the medical records. In some cases, incomplete or inconsistently documented data may have limited the precision of assessments regarding patients’ rehabilitation needs. Response bias in medical records or in patient self-report may have influenced our results. It is possible that certain groups of patients or clinicians were more likely to complete assessments or provide certain types of information, potentially skewing the findings in unmeasured ways. Therefore, refining the methods used to assess rehabilitation needs at discharge is necessary to better predict and address future needs. The psychometric properties of the NPCS have only been partly established. While the tool has shown utility in prior research, further validation studies are needed to ensure its reliability and construct validity in diverse rehabilitation populations, including trauma populations. In addition, further research is warranted to elucidate the most effective treatment interventions for reducing unmet healthcare needs and optimizing HRQoL in trauma survivors. Emotional distress is a significant long-term impact associated with HRQoL following traumatic injury [42]. We did not specifically assess the presence of anxiety, depression and PTSD and their potential effects on both HRQoL and unmet healthcare needs. This will be accomplished in an upcoming paper.

The study involved a large number of statistical comparisons. Although the main effects analyses all controlled for each other (and therefore did not need additional control for Type I error), and we applied corrections to reduce the risk of Type I error in our interaction analyses, the number of outcome tests conducted across the broader set of analyses still raises the possibility of false-positive findings. Future studies should consider using more conservative statistical thresholds or employing multivariate approaches that reduce the number of independent tests.

In conclusion, HRQoL and its associated domains remained largely stable between the 6- and 12-month follow-up assessments, apart from the ‘usual activities’ and ‘anxiety/depression’ domains, where participants reported significantly fewer problems at 12 months post-injury—consistent with findings from previous studies. Individuals with unmet healthcare needs experienced a decline in HRQoL over time, whereas those whose needs were met showed improvements. These findings underscore the essential role of timely and adequate healthcare support in facilitating recovery and improving long-term HRQoL outcomes.

## Figures and Tables

**Table 1:** Patient and injury-related characteristics, n = 446.

Category	N	%
Age, mean (SD)	51.7 (17.8)	
Sex		
Female	103	23.1
Male	343	76.9
Living status		
Living with others	287	64.3
Living alone	159	35.7
Education		
Elementary school	41	9.2
High school	196	43.9
University	191	42.8
Missing	18	4.0
Geographical location		
Central	260	58.3
Less central	186	41.7
Pre-injury ASA-PS		
Healthy	234	52.5
Systemic disease	212	47.5
Injury-related variables		
Cause of injury		
Transportation	172	38.6
Fall	194	43.5
Violence	13	2.9
Other	67	15.0
New Injury Severity Score (NISS)		
NISS ≤ 15	109	24.4
NISS > 15	337	75.6
Abbreviated Injury Scale (AIS) ≥ 3 ^1^		
AIS head ≥ 3	174	39.0
AIS face ≥ 3	11	2.5
AIS neck ≥ 3	8	1.3
AIS thorax ≥ 3	151	33.9
AIS abdomen ≥ 3	48	10.8
AIS spine ≥ 3	69	15.5
AIS upper-/lower extremities ≥ 3	71	15.9
Hospital stay		
Length of acute hospital stay (days), mean (SD)	7.7 (7.0)	
Discharge place		
Home	150	33.6
Local hospitals	193	43.3
Specialized Rehabilitation	92	20.6
Nursing home/others	11	2.5
NPCS estimates at baseline, mean (SD)		
NPCS Total	9.5 (4.9)	
NPCS Healthcare	2.6 (1.1)	
NPCS Personal care	1.8 (1.7)	
NPCS Rehabilitation	3.3 (1.7)	
NPCS Social support	1.0 (1.1)	
NPCS Environment	0.7 (1.3)	

Abbreviations: ASA-PS: American Society of Anesthesiologists Physical Status classification system; AIS: Abbreviated Injury Score; NPCS: Needs and Provision Complexity Scale. ^1^ Since a single patient may have multiple regions with an AIS ≥ 3, the total percentage could exceed 100%.

**Table 2:** EQ-5D scores at six- and twelve-month follow-ups.

EQ-5D	6 Months Mean (SD)	12 Months Mean (SD)	Paired *t*-Test *p*-Value
EQ-5D mobility	1.61 (1.0)	1.58 (1.0)	0.305
EQ-5D self-care	1.27 (0.7)	1.24 (0.7)	0.461
EQ-5D usual activities	1.99 (1.1)	1.77 (1.0)	<0.001
EQ-5D pain and discomfort	2.16 (0.9)	2.11 (1.0)	0.130
EQ-5D anxiety and depression	1.62 (0.9)	1.54 (0.8)	0.002
EQ-5D VAS	69.57 (18.9)	70.31 (20.2)	0.152

**Table 3:** Frequencies of met and unmet NPCS-Needs at six- and twelve-month follow-ups.

NPCS		6 Months N (%)	12 Months N (%)	χ² Test *p*-Value
NPCS total	Met needs	171 (38.3)	186 (41.7)	0.480
	Unmet needs	242 (54.3)	155 (34.8)	
	*Missing*	*33 (7.4)*	*105 (23.5)*	
Health and personal care domain				
Healthcare	Met needs	328 (78.8)	251 (73.0)	0.126
	Unmet needs	88 (21.2)	93 (27.0)	
Personal care	Met needs	342 (82.6)	325 (94.2)	0.137
	Unmet needs	72 (17.4)	20 (5.8)	
Rehabilitation	Met needs	196 (47.2)	229 (66.4)	0.016
	Unmet needs	219 (52.8)	116 (33.6)	
Social care and support domain				
Social support	Met needs	216 (52.2)	285 (83.1)	<0.001
	Unmet needs	198 (47.8)	58 (16.9)	
Environment	Met needs	367 (88.6)	330 (95.7)	0.535
	Unmet needs	47 (11.4)	13 (4.3)	

**Table 4:** Demographic, injury, and unmet need predictors of HRQoL trajectories across six and twelve months.

	Overall HRQOL		Mobility Problems		Self-Care Problems		Usual Activities Problems		Pain-Discomfort Problems		Anxiety-Depression Problems	
Predictor	*b*	*p*	*b*	*p*	*b*	*p*	*b*	*p*	*b*	*p*	*b*	*p*
Intercept	70.198	<0.001	1.586	<0.001	1.254	<0.001	1.867	<0.001	2.132	<0.001	1.576	<0.001
Time	0.618	0.134	−0.021	0.310	−0.008	0.550	**−0.128**	**<0.001**	−0.040	0.078	**−0.066**	**<0.001**
Man (vs. woman)	0.903	0.275	−0.049	0.258	−0.015	0.621	−0.087	0.055	−0.057	0.151	−0.056	0.129
Partnered (vs. single)	0.844	0.321	−0.035	0.430	−0.059	0.066	0.016	0.723	**0.113**	**0.006**	−0.072	0.057
Rural (vs. urban)	0.418	0.624	0.000	0.996	0.020	0.526	0.003	0.956	**0.084**	**0.041**	−0.003	0.942
Age	**−2.698**	**0.002**	**0.172**	**<0.001**	**0.108**	**0.001**	0.067	0.163	−0.013	0.751	−0.053	0.170
Injury Severity	−0.874	0.395	−0.030	0.566	0.021	0.575	0.022	0.688	**−0.114**	**0.021**	0.040	0.382
Number of Injuries	−1.168	0.249	0.007	0.897	−0.012	0.754	0.066	0.230	**0.164**	**<0.001**	**0.104**	**0.021**
Length of Stay	**−4.108**	**<0.001**	**0.211**	**<0.001**	**0.102**	**0.003**	**0.171**	**<0.001**	**0.126**	**0.004**	**0.122**	**0.003**
Unmet Healthcare Needs	**−1.904**	**0.026**	0.043	0.337	0.030	0.353	0.041	0.381	0.050	0.227	**0.086**	**0.024**
Unmet Personal Care Needs	**2.654**	**0.002**	−0.043	0.335	−0.026	0.428	**−0.102**	**0.032**	−0.074	0.074	−0.074	0.057
Unmet Rehabilitation Needs	−1.267	0.138	0.009	0.846	−0.036	0.258	0.010	0.827	0.018	0.656	−0.025	0.507
Unmet Social-Fam Needs	**−3.132**	**<0.001**	0.076	0.104	0.005	0.885	**0.145**	**0.003**	0.034	0.424	0.074	0.066
Unmet Environment Needs	−0.861	0.324	0.082	0.069	0.016	0.629	0.070	0.144	**0.084**	**0.045**	−0.005	0.903

Note. Bolded values were statistically significant at = *p* < 0.05.

**Table 5:** Demographic, injury, and unmet need predictor*time interactions on HRQOL trajectories.

	Overall HRQOL		Mobility Problems		Self-Care Problems		Usual Activities Problems		Pain-Discomfort Problems		Anxiety-Depression Problems	
Time*Predictor Interaction	*b*	*p*	*b*	*p*	*b*	*p*	*b*	*p*	*b*	*p*	*b*	*p*
*Man (vs. woman)	0.692	0.096	−0.019	0.371	0.013	0.320	−0.031	0.190	−0.051	0.027	−0.011	0.564
*Partnered (vs. single)	−0.471	0.270	−0.026	0.218	−0.001	0.942	0.024	0.323	0.037	0.123	0.031	0.128
*Rural (vs. urban)	0.108	0.799	0.042	0.047	−0.008	0.568	−0.002	0.920	0.019	0.418	0.013	0.510
*Age	−0.552	0.206	0.030	0.176	0.013	0.341	0.021	0.387	0.026	0.284	0.021	0.311
*Injury Severity	−0.251	0.631	0.027	0.302	0.015	0.355	−0.020	0.488	−0.019	0.501	−0.011	0.653
*Number of Injuries	0.676	0.195	−0.043	0.102	−0.006	0.724	−0.051	0.082	−0.063	0.028	−0.024	0.330
*Length of Stay	−0.914	0.050	−0.005	0.842	0.013	0.367	0.015	0.584	0.018	0.494	−0.005	0.813
*Unmet Healthcare Needs	**−1.251**	**0.004**	−0.005	0.812	0.021	0.118	−0.044	0.076	−0.016	0.519	−0.010	0.639
*Unmet Personal Care Needs	0.011	0.979	0.007	0.739	0.014	0.280	−0.002	0.942	−0.019	0.421	0.023	0.259
*Unmet Rehabilitation Needs	−0.337	0.426	−0.007	0.759	−0.015	0.267	−0.004	0.856	0.011	0.641	−0.003	0.864
*Unmet Social-Fam Needs	0.023	0.959	−0.017	0.452	−0.016	0.226	−0.012	0.627	0.006	0.817	−0.013	0.542
*Unmet Environment Needs	0.569	0.201	−0.021	0.352	0.012	0.424	0.024	0.351	−0.003	0.919	0.025	0.240

Note. Bolded values were statistically significant with the Holm–Bonferroni correction starting at α = 0.05. Intercept, main effects, and time were also included but are excluded from the table for simplicity. * denotes predictor × time interaction.

## Data Availability

The datasets generated and/or analyzed during the current study are not publicly available but are available from the corresponding author on reasonable request.

## Supplementary Materials

The following supporting information can be downloaded at https://www.mdpi.com/article/10.3390/jcm14124213/s1, Table S1: 95% confidence intervals of parameter estimates from main effects models; Figure S1. EQ-5D domain scores (mean) at six- and 12-months post-injury; Figure S2. Proportions of met and unmet NPCS needs at six- and 12-months follow-ups, based on the total score.
