# Supplementary material for: Health-Related Quality of Life and Unmet Healthcare Needs in the First Year Following Moderate-to-Severe Traumatic Injuries—An Observational Study
Source: J Clin Med. 2025 Jun 13;14(12):4213. doi: 10.3390/jcm14124213 (PMC12194146; doi:10.3390/jcm14124213)
Supplement: Supplementary file 1 [file jcm-14-04213-s001.zip › jcm-3644329-supplementary.pdf]

Table S1: 95% confidence intervals of parameter estimates from main effects models

	**Overall HRQOL**		**Mobility Problems**		**Self-Care Problems**		**Usual Activities Problems**		**Pain-Discomfort Problems**		**Anxiety-Depression Problems**	
Predictor	Lower	Upper	Lower	Upper	Lower	Upper	Lower	Upper	Lower	Upper	Lower	Upper
Intercept	68.572	71.810	1.503	1.669	1.193	1.312	1.779	1.956	2.056	2.209	1,504	1,648
Time	-0.175	1.444	-0.061	0.019	-0.032	0.018	-0.173	-0.082	-0.087	0.003	-0.105	-0.029
Man (vs. woman)	-0.704	2.592	-0.15	0.019	-0.084	0.038	-0.184	-0.004	-0.145	0.011	-0.124	0.022
Partnered (vs. single)	-0.715	2.668	-0.129	0.043	-0.129	-0.006	-0.078	0.107	0.028	0.188	-0.155	-0.005
Rural (vs. urban)	-1.570	1.811	-0.084	0.089	-0.043	0.081	-0.074	0.11	-0.001	0.159	-0.072	0.079
Age	-4.516	-1.112	0.098	0.274	0.059	0.184	-0.021	0.166	-0.085	0.077	-0.121	0.031
Injury Severity	-2.719	1.298	-0.128	0.076	-0.049	0.097	-0.088	0.13	-0.218	-0.028	-0.061	0.117
Number of Injuries	-3.150	0.876	-0.093	0.112	-0.081	0.065	-0.038	0.18	0.072	0.263	0.012	0.191
Length of Stay	-6.391	-2.837	0.146	0.327	0.037	0.166	0.101	0.294	0.068	0.236	0.05	0.208
Unmet Healthcare Needs	-2.241	1.236	-0.067	0.113	-0.019	0.111	-0.056	0.134	-0.085	0.081	-0.06	0.095
Unmet Personal Care Needs	1.110	4.568	-0.223	-0.047	-0.162	-0.035	-0.234	-0.046	-0.19	-0.027	-0.134	0.02
Unmet Rehabilitation Needs	-2.531	0.967	-0.078	0.1	-0.084	0.045	-0.09	0.1	-0.057	0.108	-0.083	0.072
Unmet Social-Fam Needs	-4.636	-1.060	-0.044	0.138	-0.067	0.062	-0.015	0.18	-0.055	0.113	-0.001	0.157
Unmet Environment Needs	-2.228	1.457	-0.022	0.165	-0.07	0.069	-0.084	0.116	-0.002	0.173	-0.08	0.084

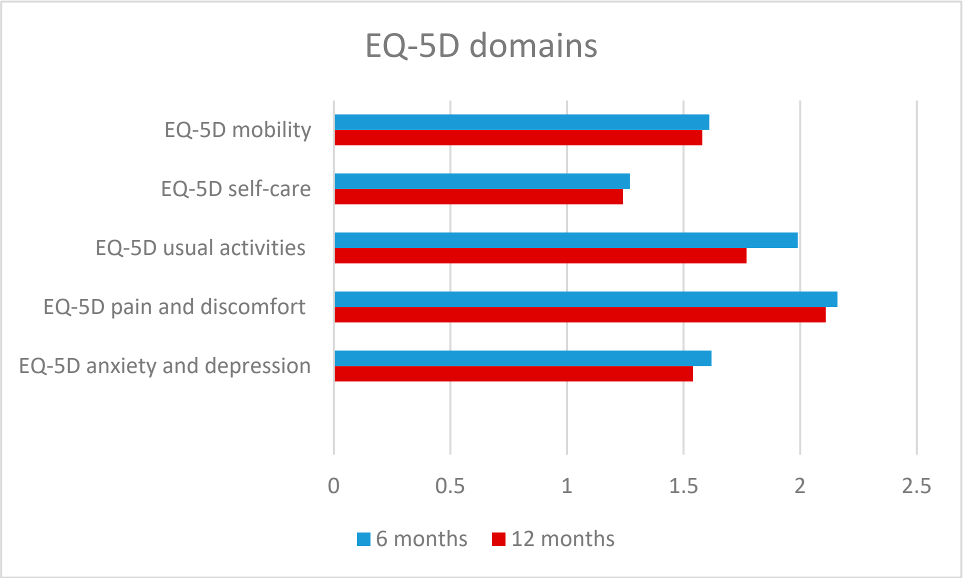

Figure S1. EQ-5D domain scores (mean) at six- and 12-months post-injury.

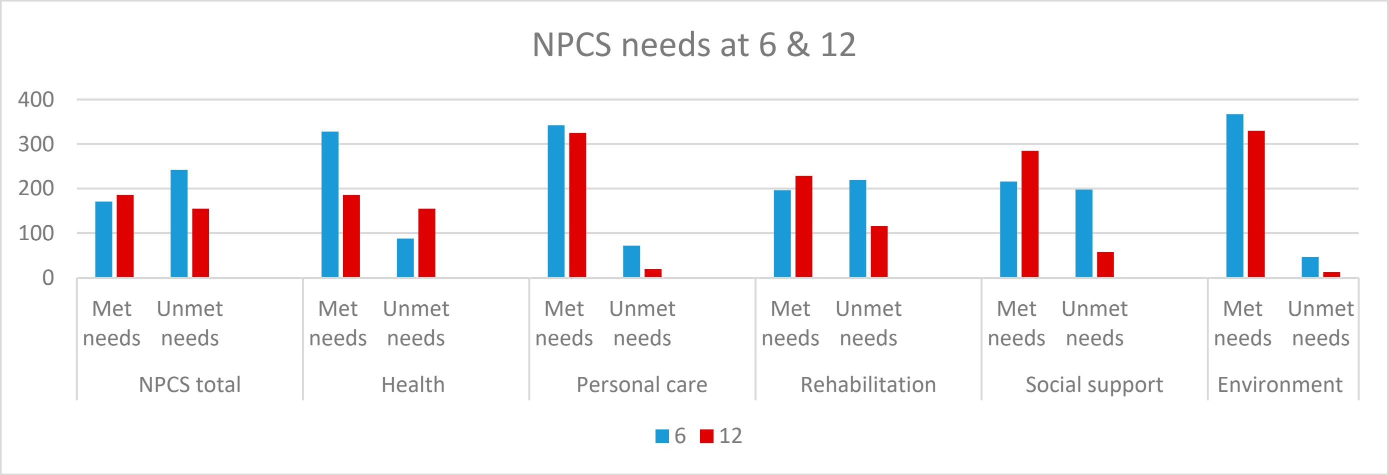

Figure S2. Proportions of met and unmet NPCS needs at six- and 12-months follow-ups, based on the total score
